# A role for zinc in the mitigation of zoonotic disease attributable to RNA viruses—a review with application to One Health perspective

**DOI:** 10.1093/af/vfaf033

**Published:** 2025-10-14

**Authors:** Christopher D Ashworth, Arturo Gomez, Michael T Socha, Mark E Branine

**Affiliations:** Zinpro Corporation, Eden Prairie, MN; Zinpro Corporation, Eden Prairie, MN; Zinpro Corporation, Eden Prairie, MN; Zinpro Corporation, Eden Prairie, MN

**Keywords:** nutritional immunity, One Health, RNA virus, zinc, zoonotic disease

**Implications**

- One Health is a concept that serves as a common foundation for connecting human and animal populations, primarily those of domestic livestock, poultry, wildlife, and companion animals, with environmental and socioeconomic factors, as it relates to the understanding, prevention, and treatment of multiple zoonotic diseases that can afflict all groups on a global basis.
- Numerous zoonotic diseases affecting both human and animal populations are attributable to various families of RNA viruses as the primary etiologic agent.
- A growing body of research is providing evidence to support the concept that dietary zinc can potentially have a role in the prevention and mitigation of zoonotic diseases attributable to RNA viruses.
- The increasing risk to One Health from an increased prevalence of RNA viral diseases requires a better understanding of the role nutritional immunity may have for prevention and mitigation.

## Introduction

The concept of One Health may be associated with many diverse interpretations in both scope and practice as viewed from a particular frame of reference or perspective. From a general perspective of human and veterinary medicine, One Health is based on the general premise that human and veterinary medicine are connected at multiple levels, with environmental and socioeconomic factors often providing a common denominator. The interdisciplinary One Health High-Level Expert Panel (OHHELP; Adisasmito et al., 2022) has broadly defined One Health as an integrated, unifying approach that aims to sustainably balance and optimize the health of people, animals, and ecosystems. The OHHELP definition also recognizes health of humans, domestic and wild animals, plants, and the wider environment (including ecosystems) constitutes an often-diverse set of factors that are intimately connected, integrated, and interdependent at multiple points and levels. Multiple events are occurring to converge many disparate effects into a generalized synergism affecting the health of global human and animal populations. These effects may include growing numbers of human populations that are expanding into new geographic areas, either through population growth, conflict-induced migration, or ecosystem disruption as affected by climate change. These factors along with increased international trade and global travel may result in a closer proximity between people and animals (wild and domestic) that allows for increased passage of pathogens that may facilitate the potential dissemination and emergence of existing and novel zoonotic diseases (Keusch et al., 2022). The recent occurrence of global pandemics, including severe acute respiratory syndrome-associated coronavirus infection (SARS-COVID) in 2019 and the increasing prevalence of the H5N1 strain of highly pathogenic avian influenza A (HPAI-A) have required increased attention to the role of viral pathogens and specifically RNA viruses may have and their implications on One Health. A recent review by Ameni et al. (2024) documented seven major zoonotic outbreaks attributable to RNA viruses from 1997 to 2021 indicating three primary causative groups of RNA viruses associated with these outbreaks were: (1) HPAI-A and swine-origin influenza (H1N1); (2) SARS-COVID-19, Middle East respiratory syndrome (MERS-COVID), and other Coronavirus borne diseases; and (3) Zika virus infections. RNA viruses are also responsible for causing rabies, foot and mouth disease (FMD), and a myriad of other diseases affecting both human and/or animal populations. In general, viral diseases, including those attributable to RNA viruses, have had profound impacts on human and veterinary morbidity, mortality, and socioeconomic loss (Ameni et al., 2024).

Given the potential for RNA viruses to rapidly mutate, enabling them to spread from a primary host to other species, consideration needs to be focused on alternative methods to restrict further cross-species transmission and potential alternative methods for prophylaxis and therapy. Similarly, because of the difficulty, time, and cost associated with developing antiviral pharmacologic interventions, consideration for nutritionally based or other alternatives is required. Recently, this has become more urgent with the documented presence of the HPAI-A virus being found in dairy cows with several confirmed cases of this virus being transmitted to humans as well as 400 other avian and mammalian species (Center for Disease Control [CDC], 2025). Similarly, the specter of potential future pandemics being caused by novel and yet unrecognized RNA viruses remains on the horizon.

An in-depth discussion of RNA virology and the research concerning basic or mechanistic aspects of zinc on viral physiology and associated immunology is beyond the scope of this paper. The research in this area has been extensive and globally based and the subject for several excellent reviews, which will be highlighted throughout this paper. Rather, our objective is to provide an overview of how the general concept of nutritional immunity may have application with particular attention on published research investigating the effects of zinc on RNA viral biology and its potential application for mitigating the risk that RNA viral diseases pose to both human and animal populations from a One Health perspective.

### Concepts of nutritional immunity and metallopharmaceutics as applied to trace metals

Trace elements (TE) are those provided in the diet at rates of mg or ng/kg of dry matter (DM) intake and include metals such as iron, zinc, manganese, copper, selenium, and chromium, among others, and have numerous biological and physiological roles essential for all recognized life forms, including bacteria (Shenkin, 2006). The critical requirement for TE by bacteria thereby underscores the basic premise of nutritional immunity as it is presently understood. The concept of nutritional immunity has classically referred to the ability of an animal host to restrict access of nutrient metals from bacteria as a means of helping to mitigate pathogenesis. Murdoch and Skaar (2022) discussed the concept of nutritional immunity as the battle for TE at the host–pathogen interface. The traditional model (Kehl-Fie and Skaar, 2010; Hennigar and McClung, 2016; Murdoch and Skaar, 2022) for nutritional immunity has been where a host employs various metabolic strategies to limit the access of iron to a bacterial pathogen infecting the body. As described by Murdoch and Skaar (2022) in addition to other immunological responses following infection by a bacterial pathogen, the affected host restricts the amount of iron potentially available to the bacterial pathogen through a systemic reprogramming of iron homeostasis sequestering iron into many cells, such as macrophages, hepatocytes, and enterocytes. Concurrently, iron absorption from the gut is reduced with the combined effects being an overall reduction in available iron to support bacterial growth. In addition to iron, similar nutritional immunity mechanisms for limiting bacterial access to manganese and zinc are also activated in response to invasion by a bacterial pathogen (Kehl-Fie and Skaar, 2010).

Like the role of TE in the overall defense against bacterial infection, whether through nutritional immunity or a generalized systemic immune response, TE has also played a therapeutic role in medicine throughout history. Lemire et al. (2013) have reviewed the critical role of various TE as metallopharmaceutics in the treatment of numerous diseases, especially prior to the discovery of antibiotics. The discovery and development of Salvarsan, an organo-metallic complex of arsenic, for the treatment of syphilis by Paul Erlich and coworkers in 1910 ushered in the age of chemotherapy as a vital component in both human and veterinary medicine. However, the general concepts of both nutritional immunity and metallopharmaceutics to this point have primarily involved their application to bacterial pathogens and not viral-genic disease. As will be discussed in the remainder of this paper, the concepts of nutritional immunity and metallopharmaceutics as applicable to viral-based disease, while valid, may need to be approached from a different perspective than that generally applied to bacterial pathogens. For this purpose, the discussion will focus on zinc as a potential intervention for aiding in the mitigation of zoonotic disease caused by RNA viruses.

#### Basic facts on zinc as a dietary nutrient.

Zinc was first identified as essential for human nutrition in the 1960s (Prasad, 2012). Research studies conducted throughout the intervening years have documented the numerous roles zinc has in many metabolic, physiological, structural, and immunological processes (Maret, 2025). As a transition metal, zinc has several intrinsic properties that make it a vital component or cofactor in over 3,000 known metabolic processes and is second only to calcium in this regard (Singh and Prasad, 2023; Maret, 2025). A dietary supply of zinc is required daily since little is stored in a form that can be readily used to support physiological or immune functions (Huang et al., 2025). Unfortunately, it has been estimated that approximately two billion people, primarily those living in less developed countries may be deficient in zinc; although, other groups in more developed countries such as the elderly or those with lesser socioeconomic status may similarly have inadequate or marginal daily dietary zinc intake (Prasad, 2012; Wessels and Brown, 2012). A similar situation may exist for domestic food animal populations, where adequacy of zinc status and dietary sufficiency remains difficult to assess in many circumstances and likely contingent on dietary levels as affected by geographic, environmental, and economic factors. This may be especially relevant for non-supplemented livestock grazing pasture or rangeland where zinc and other nutrient concentrations in the plants and forage may be marginal and vary across the year (Brummer et al., 2019; Spears et al., 2022). Similarly, monogastric livestock and poultry may be at greater risk for lower zinc absorption due to the presence of dietary phytates (Herdt and Hoff, 2011). Assessing functional zinc status in both humans and animals, however, remains problematic as its measurement is subject to multiple factors and lack of validated methods (Franco et al., 2024; Maret, 2025); however, general categories based on serum levels and(or) dietary intake concentration are used for assessing adequate, deficient, or marginal status in human and animal populations (Herdt and Hoff, 2011; Maret, 2025). Numerous chemical sources of zinc are typically available for dietary supplementation. Inorganic as well as organic sources can vary significantly in their overall bioavailability. Inorganic zinc sources such as zinc oxide are generally less bioavailable than sulfate or chloride forms. Organic forms such as those where zinc is complexed to an organic ligand such as amino acid may be regarded as generally more available for absorption (Wedekind et al., 1992). Developing nutritionally based strategies for potential mitigation of RNA viral disease will require consideration of both dietary zinc source and level across both at-risk human and animal populations. The application of molecular modeling described by Choudhury et al. (2021) may be a potential avenue for facilitating further research.

#### Relationship of zinc and immune function.

Gombart et al. (2020) reviewed the role of micronutrients in immune function. Specifically, zinc has a key role in over 100 metabolic systems and is integral to all aspects of immune function including those related to viral diseases, and especially those attributable to RNA viruses. An abridged summary of the role of zinc in various immune functions is shown in Table 1 (Gombart et al., 2020). Zinc has a wide diversity of roles through its involvement in many different biological processes including cell proliferation and differentiation, cell cycle activities, reactive oxygen species (ROS) homeostasis, and immune response (Read et al., 2019; Gombert et al., 2020). Zinc is involved in improving response of immune cells to a virus, as well as being involved in zinc-dependent protein production that kill viruses, directly preventing RNA viruses from replicating (Ibs and Rink, 2003; Gao et al., 2018). Zinc reduces inflammatory cytokines and chemokines such as tumor necrosis factor (TNF-α), interleukins (IL-1β, IL-6), monocyte chemoattractant protein-1 (MCP-1), vascular cell adhesion molecule (VCAM), and C-reactive protein (CRP) as well as oxidative stress markers such as lipid peroxidation products and DNA intermittent oxidation products (Daskou et al., 2023). Other research has shown that zinc is crucial in regulating inflammatory cytokines by reducing IL-6 concentrations and proinflammatory responses via kappa-light-chain-enhancement of activated B cells (NF-kB), alleviating redox-induced cellular injury controlling oxidative stress, and avoiding host-tissue damage by inflammation (Jarosz et al., 2017; Wu et al., 2019).

**Table 1.** Summary of zinc in immune function^a^^,^^b^

Immune function	Zinc function or activity	Reference
Immune cell function	Maintains/enhances natural killer cell activity Role in immune cell growth and differentiation Enhance phagocytic activity of macrophages Improves phagocytic activity of monocytes	Maggini et al., 2018; Gao et al., 2018; Wu et al., 2019
Inflammation and antioxidant effects	Anti-inflammatory agent Modulation of cytokine release Dampening development of proinflammatory Th17 and Th9 cells Influence of cytokine generation (Il-2, Il-6, TNF-α) Antioxidant protection from ROS Influences activity of antioxidant proteins	Jarosz et al., 2017; Wu et al., 2019; Wessels and Rink, 2019
T-cell function	Induces proliferation of T-cells Supports Th1 cytokine production and response Required for T-cell development, differentiation, and activation Development of Treg cells and maintenance of immune tolerance	Wu et al., 2019
Antibody production/development	Involved with antibody production (IgG)	Ibs et al., 2003
Antigen response	Involved with antibody response to antigens Involved in maintaining immune tolerance	Wu et al., 2019

^a^Summarized from Gombart et al. (2020).

^b^Abbreviations: IgG = immunoglobin type G; ROS = reactive oxygen species; Th = T helper cells; Il = interleukin; TNF-α = Tumor necrosis factor alpha; Treg = T regulatory cells.

Another aspect of the immune response to viral infection concerns the response of natural killer cells (NK). This population of immune cells are a class of lymphoid cells that exhibit an absence of T-cell receptor on their cell surface and participate in the innate immune response having an essential role in immunity against microbial infections with the ability to kill virus-invaded cells (Daskou et al., 2023). These cells eradicate viruses or virus-infected cells by three main mechanisms: (1) the excretion of interferon (IFN-γ), an antiviral cytokine; (2) secretion of cytolytic granules containing granzyme and perforin; and (3) through use of death-receptor-mediated apoptosis (Read et al., 2019; Sadeghsoltani et al., 2022). It is known that aberrant modulation of NK cell functions is in part impacted by an activated cytokine storm during a virus attack, which is highly linked to the severity of SARS-COVID-19 (Heydarazadeh et al., 2021). Thus, enhancement of NK function would provide a positive effect on viral clearance in the body. Amling et al. (2025) reported zinc supplementation positively modulated NK function, whereas zinc deficiency reduced NK activity; an effect that was reversed when zinc was supplemented to cells. Zinc insufficiency has depressed the cytolytic activities of NK through a decreased recognition of the major histocompatibility complex-1 molecule (MHC). Zinc supplementation up-regulated cytolytic activity of NK cells and cytotoxic T-lymphocytes (CD8^+^) toward the infection site (Daskou et al., 2023).

### Role of zinc for prevention and mitigation of diseases caused by RNA viruses

Viral RNA-dependent RNA polymerase (RdRp) is an internal catalytic enzyme required for RNA synthesis from the viral RNA template of this family of viruses. This enzyme is a critical, nonstructural protein required for the viral replication life cycle and subsequent amplification of infection without the DNA synthesis stage. In vitro research has shown inhibitory effects of zinc on SARS-COVID virus by its binding to the active aspartic acid triad and other binding sites that can destabilize RdRp or interfere with the fidelity-check mechanism (Zoghi et al., 2021). Similar effects of zinc occur by inhibiting viral replication cycle, RNA synthesis, and protein processes in several diverse viral infections including human immunodeficiency virus-1 (HIV-1), hepatitis C virus, coronavirus, and varicella-zoster virus have been reported (Read et al., 2019; Prasad et al., 2022). Several recent reviews have provided information to suggest zinc status is a contributor affecting the severity of SARS-COVID-19 and that optimizing dietary zinc may be effective for mitigation and/or treatment of this disease (Arentz et al., 2020; Skalny et al., 2020; Wessels et al., 2020; Sadeghsoltani et al., 2022; Mouchati et al., 2024). In their summary of multiple research studies, Read et al. (2019) have suggested the potential role of zinc as an antiviral agent may be classified into two categories: (1) zinc supplementation implemented to improve the antiviral response and systemic immunity in patients with zinc deficiency, and (2) zinc treatment performed to specifically inhibit viral replication or infection-related symptoms.

### Potential mechanisms for antiviral response of zinc

Several publications have reported research investigating potential mechanisms for antiviral effects of zinc (Kaushik et al., 2017; Read et al., 2019; Heydarazadeh et al., 2021; Zoghi et al., 2021; Prasad et al., 2022; Sadeghsoltani et al., 2022; Daskou et al., 2023; Kumar et al., 2023). Directly assembling functional RNA strains is unique to RNA viruses as from all other organisms, which use DNA as the template for transcription of RNA. Such RdRp-dependent synthesis allows for a faster and easier replication process for RNA viruses compared with DNA viruses. Targeting RdRp would provide a potential therapeutic strategy for treatment of SARS-COVID-19 and other RNA viruses as this enzyme is needed to signal the original RNA strand to replicate (Kaushik et al., 2017; Zoghi et al., 2021). Zinc may inhibit the viral replication cycle of coronavirus in SARS-COVID-19 by targeting RdRp with this response being more efficacious to a greater concentration of dietary zinc (Aftab et al., 2020; Heydarazadeh et al., 2021). Moreover, pyrithione, a zinc ionophore, has been shown to inhibit the replication cycle of SARS-COVID-19 in vitro when in the presence of low-level zinc (2–8 µM), suggesting zinc can penetrate the viral membrane and suppress the viral replication cycle by targeting viral RNA polymerase (Heydarazadeh et al., 2021). Other potential benefits from zinc are reduction of plasma CRP concentration downregulating the response to inflammation, decreased recombinant human angiotensin-converting enzyme (ACE) activity and expression of modulating effect on SARS-COVID/ACE-2 interaction.

Other research has indicated pregnant female patients with SARS-COVID-19 infection have a reduced concentration of serum zinc during pregnancy compared with noninfected pregnant women serving as controls (Wessels and Brown, 2012; Consales et al., 2024). Another prospective study revealed that SARS-COVID-19 patients have a significantly decreased serum concentration of zinc when compared with a noninfected control group (serum zinc: 74.5 µg/dL vs. 105.8 µg/dL, respectively; Wessels et al., 2020). Serum zinc concentrations of 89 µg/dL or less have been considered zinc deficient, while 90 µg/dL or more is considered zinc sufficient or normal in the human body (Wessels et al., 2020). Moreover, zinc deficiency has been found to be correlated to the severity of SARS-COVID-19 infection as indicated by higher rates of complications, such as acute respiratory distress, the need for ancillary steroid therapy administration, prolonged duration of hospitalization, and death. These findings may explain why elderly populations, who are more susceptible to zinc insufficiency, have a significantly higher risk of SARS-COVID-19 morbidity and mortality (Wessels et al., 2020).

### Potential roles for zinc to mitigate disease in food-producing animals

As reviewed above, an increasing body of experimental and clinical research studies are providing evidence that zinc has an important role in antiviral immunity and has significant antiviral properties. The antiviral effects of zinc seem to be virus-specific with a great diversity of RNA viral families that affect different tissues, organ systems, and species being susceptible (Sumbria et al., 2019). The 2023–2025 outbreak of HPAI-A provides an example of how this virus has spread from wild bird populations to domestic flocks of chickens and turkeys to many other avian species, terrestrial, and aquatic mammalian species (including humans) resulting in widespread morbidity and mortality (CDC, 2025). In Table 2 some common human and animal RNA viruses as etiologic agents in zoonotic disease are listed (Rahman et al., 2020).

**Table 2.** Examples of RNA viral zoonotic diseases^a^

Disease	Viral agent	Primary host species	Affected species	Human symptoms
Avian influenza	*Influenza A virus* Alphainfluenzavirus Orthomyxoviridae	Avians species such as migratory waterfowl	Ducks, chickens, turkeys, dogs, cats, pigs, whales, horses, seals, cattle, sheep, and wild birds; humans	Flu-like symptoms, diarrhea, pneumonia, respiratory
Rabies	Rhabdoviridae lyssavirus	Bats—primary mammalian carnivores	Cattle, horses, cats, dogs, bats, monkeys, wolves, skunks, rabbits, and coyotes; humans	Nervous system disorders. Generally fatal
Severe acute respiratory syndrome (SARS)	*SARS coronavirus* Genus—Coronavirus Family—Coronaviridae	Bats Masked palm civets	Bats, dogs, cats, ferrets, minks, tigers, and lions; humans	Influenza-like symptoms, fever, muscle pain, and severe cases progressing to a respiratory disease and pneumonia
Hantavirus pulmonary syndrome	*Hantavirus* Genus—Orthohantavirus Family—Hantaviridae	Bats Rodents (deer mice, cotton rats, rice rats, white-footed mice, shrews, and moles)	Humans	Respiratory problem, high fever, dizziness, chills, and abdominal problems. Can be fatal
West Nile fever	*West Nile virus* Genus—Flavivirus Family—Flaviviridae	Birds—primarily migratory birds including corvids and sparrows	Horses, birds, reptiles, humans	Headache, skin rash, swollen lymph nodes, stiff neck, disorientation, coma, tremors, convulsions, and paralysis
Ebola Hemorrhagic Fever	Ebola virus Genus—Ebolavirus Family—Flaviviridae	Primates including old world monkeys, gorillas, chimpanzees, apes, and wild antelopes	Humans	Fever, intense weakness, muscle pain, headache, sore throat, hemorrhage, vomiting, diarrhea, and kidney and liver failure leading to death

^a^Adapted from Rahman et al. (2020).

The research discussed to this point has been related to more basic aspects of zinc as an antiviral with an emphasis toward human medicine. However, the zoonotic potential for many RNA viruses to cross-affect multiple species remains a viable concern. Sadeghsoltani et al. (2022) have suggested influenza viruses, respiratory syncytial virus, and SARS-COV2 are among the most dangerous respiratory viruses to both human and animal populations. In addition to RNA respiratory viruses, the RNA picornovirus responsible for FMD remains an ongoing threat to livestock production across the globe. Polatnick and Bachrach (1978) published research indicating significant inhibitory effects of zinc on this virus using a cell culture model. These researchers indicated that zinc was the most efficacious of the chemical agents evaluated and mediated the observed inhibition by interfering with the cleavage required to activate FMD-specific proteins. More recently, Lee et al. (2024) have reported on their research using oral zinc sulfate administration as an adjuvant for inactivated FMD vaccines. Their results from serological analyses on mice and pigs, measuring secretory IgA levels and evaluating the expression of mucosal immunity-related genes in pigs indicated that zinc produced a more sustained and enhanced long-term immune response by effectively stimulating mucosal and overall systemic immunity. A practical application of these results could reduce the frequency of required FMD vaccinations or allow for a decreased antigen dose. Globally, FMD outbreaks can occur in various countries where it is not endemic, causing economic devastation and substantial economic loss. Optimistically, areas with no presence of FMD will remain free from an outbreak; however, the threat of this occurring whether by an accidental introduction or by an act of bioterrorism remains a viable concern.

### Environmental and socioeconomic impacts from RNA viral disease—a One Health perspective

For human society, the recent 2020 SARS-COVID pandemic provides a relevant example of the high-cost RNA viral disease may cause in terms of mortality, morbidity, economic loss, and overall pain and suffering experienced by individuals, families, and nations. In food animal production, biosecurity protocols for controlling outbreaks of RNA viral diseases such as FMD and HPAI may involve vaccination and quarantine of affected premises that under some circumstances may involve depopulation of animals by mass euthanasia and consequent disposal of animals exposed to the virus. (WOAH, 2012; CDC, 2025). The immense quantities of biomass resulting from depopulation require disposal in landfills or by other methods can present significant environmental challenges. The outbreak of HPAI in poultry has necessitated euthanasia of millions of chickens (CDC, 2024). The spread of HPAI in dairy cows has resulted in significant increases in culling and significant reductions in milk production (CDC, 2025). An outbreak of FMD in geographic areas of major swine and cattle production would likewise have critical economic and logistical consequences resulting from the mass depopulation and disposal of affected and exposed animals (WOAH, 2012). Lastly, the spread of HPAI into natural wildlife populations resulting in severe mortality and morbidity of numerous species will have significant impacts across multiple ecosystems (Rahman et al., 2020; CDC, 2024).

The development of effective strategies such as providing supplemental dietary zinc either as a metaphylactic, prophylactic, or therapeutic intervention could potentially mitigate economic loss associated with decreased productivity and reductions in the numbers of animals requiring depopulation by improving resilience to viral infection as mediated by enhancing immune response to reduce viral load and transmissibility and/or improved efficacy of vaccination. Additionally, given the role of various RNA viruses in the etiology and pathogenesis of disease in poultry and livestock production, similar benefits would accrue through improvements in production efficiency and economics and, as importantly, the health and well-being of the animal. Improving the mitigation of RNA viral infection in animal populations could also reduce the spread of zoonotic disease to at-risk human and other animal populations as well.

## Summary

The concept of One Health has helped provide the framework for a synthesis of those issues and concerns affecting both human and veterinary medicine and potential consequences on associated environmental and socioeconomic issues. As discussed in this paper, the potential application of nutritional immunity as an alternative to more traditional pharmacologic interventions may have merit for contributing to improving overall global health outcomes in human populations as well as those of livestock, poultry, and other animals. Similarly, a large and expanding body of research is helping provide an evidence-based foundation for zinc as an integral mediator between potential infection of human and(or) animals by an RNA virus and the ability of the host’s immune system to effectively resist pathogenic invasion and reduce viral load. The application of this information will, however, require a rethinking of nutritional immunity as it currently stands. Whereas nutritional immunity has traditionally required the sequestration of trace minerals to effectively starve a bacterial pathogen (Hennigar and McClung, 2016), its application to RNA viruses may require strategic supplementation of zinc (and perhaps other micronutrients). This will require additional knowledge and insight into how to balance dietary zinc levels and sources to achieve optimum benefits. Development of improved methods to better assess functional zinc status in normal and at-risk human and animal populations is required, as are the establishment of cross-functional databases to facilitate the assessment and definition of zinc adequacy and status. Further research requires both basic and mechanistic approaches, suggesting viral challenge studies to evaluate the response of differing levels and sources of dietary zinc, as well as larger-scale longitudinal research with targeted populations of people, livestock, and poultry to provide additional data regarding the potential efficacy of zinc to achieve a desired outcome and answers to questions yet remaining.

## About the Authors

**Christopher D. Ashworth** is currently a research veterinarian with Zinpro Research and Discovery group. Dr. Ashworth studied dairy science at Louisiana State University and went on to graduate from Louisiana State University School of Veterinary Medicine. He completed a large animal medicine and surgery internship at the University of Illinois at Urbana-Champaign and a three-year residency in food animal medicine and surgery, becoming a specialist in diseases of food animals. Dr. Ashworth’s extensive dairy and beef industry experience spans over 25 years with some of the most recognized companies in the animal health industry. His experience includes academic, nutritional, and infectious disease research and genetics research, as well as being extensively involved with animal welfare. In 2017, Dr. Ashworth joined the Zinpro Research and Discovery team and is responsible for working with dairies, cow/calf, and feedlots on a global basis providing technical information and consultation. Dr. Ashworth is a well-regarded speaker addressing many topics related to health issues and diseases affecting dairy and beef cattle. Dr. Ashworth has served as an officer and member for several professional veterinary organizations. Dr. Ashworth also owns and manages a 250-head beef cow calf operation. Corresponding author: cashworth@zinpro.com

**Arturo Gomez** earned a veterinary medicine degree from the University of Santiago de Compostela in Spain. He began his professional career as a bovine practitioner in Spain and Portugal. He attended the University of Wisconsin-Madison, where he completed a residency program in Food Animal Production Medicine and a master’s degree in clinical investigations. Dr. Gomez completed a PhD in public health and epidemiology from the University of Wisconsin School of Veterinary Medicine in 2014. Following the completion of his doctorate Dr. Gomez began his employment with Zinpro Corporation as a dairy veterinarian. Currently, Dr. Gomez is a research veterinarian and global ruminant group manager for Zinpro Research and Discovery providing nutritional services and overseeing research in Europe, Northern Africa and the Middle East. Dr. Gomez is a recognized global expert on many health issues affecting dairy cattle with special emphasis on digital dermatitis.

**Michael Socha** has had a 25+ year career with Zinpro Corporation, which has entailed numerous roles providing technical and nutritional services to a global dairy cow clientele. Since 2016, Dr. Socha has served as Associate Director for Zinpro’ s Research and Discovery group, where he directs research and product development activities for livestock, poultry, aquaculture, and companion animals. Dr. Socha received his BS and MS degrees in dairy science at the University of Wisconsin and his PhD in animal and dairy sciences from the University of New Hampshire. His doctoral research focused on determining methionine requirements of lactating dairy cows, while his MS thesis analyzed the effect of feeding heated soybeans to early lactation cows on milk production, composition, and fatty acid composition. Throughout his career, Dr. Socha has published numerous research papers. Dr. Socha directs an international group of nutritionists, veterinarians, medicinal chemists, and technicians focused on conducting evidence-based research and development of novel solutions addressing a myriad of nutritional and health concerns across the food animal industry.

**Mark Branine** is a ruminant nutritionist with a 45-year career managing clinical, product, and technical development research projects to support improvements for beef cattle nutrition, health, and management. He holds a doctorate in ruminant nutrition from New Mexico State University with MS and BS degrees in animal science from the University of Wyoming and Colorado State University, respectively. He has recently retired as a research nutritionist from Zinpro Corporation.
